# Frank Day, Konkow Maidu (1902–1976). The Water Test (c. 1970–1975).

**DOI:** 10.3201/eid0904.AC0904

**Published:** 2003-04

**Authors:** Polyxeni Potter

**Affiliations:** * Centers for Disease Control and Prevention, Atlanta, Georgia, USA

**Figure:**
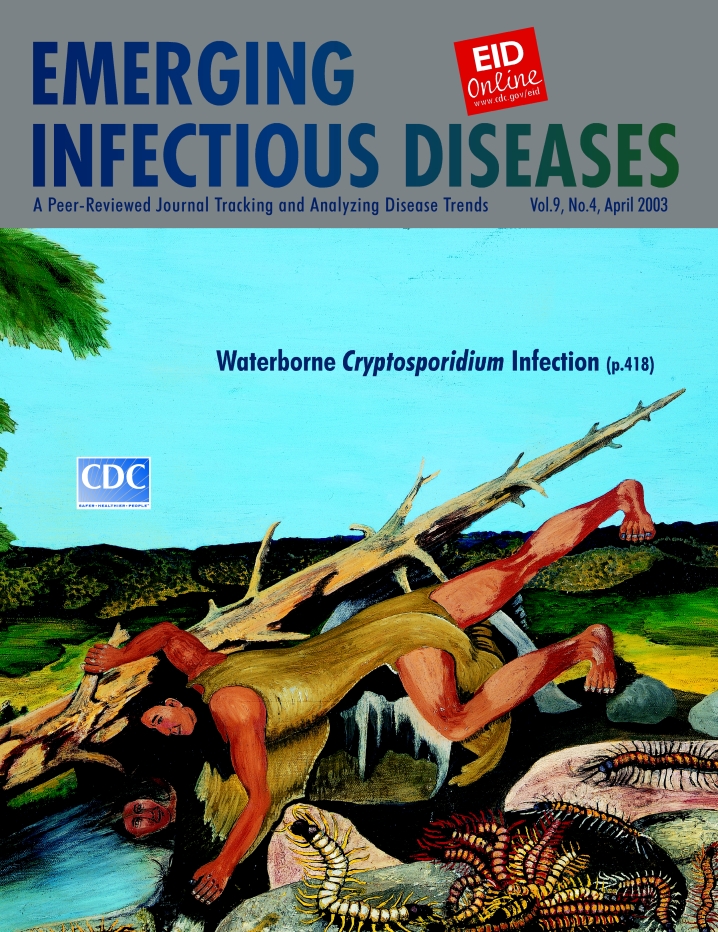
Oil on canvas, 48.26 cm x 62.86 cm. Heard Museum, Phoenix, Arizona, USA.

“Once in while I take up color and paint a little bit because if I do not do this, all things will be forgotten,” Frank Day said of his work. The artist, who was born into the Konkow Maidu tribe in Berry Creek, California, was concerned that if not documented, his tribe’s perception of the world would disappear (*1*). A gifted storyteller and teacher as well as talented artist, Day translated this tribal perception of the world into narrative images filled with Maidu themes in bold color.

Like many Native Americans of his generation, Day was under pressure throughout his life to abandon Native cultural practices in the interest of assimilation. A boarding-school student, he grew up wearing a standard school uniform and learning the ways of the broader society. But after the death of his father in 1922, Day set off to explore the history, language, ceremonies, and customs he had learned from him and other tribal elders. For a decade, he traveled western areas that had been inhabited by Indian tribes for hundreds of years and finally settled in California, where he worked as an agricultural laborer. After a serious injury, he turned to art as therapy. Without formal training, he soon exhibited untapped artistic talent and pure, distinctive style (*1*).

In the more than 200 canvasses he painted in the last two decades of his life, Day integrated myth, legend, and oral tradition into powerful compositions. His paintings, created from memory rather than observation, had a dreamy, symbolic, and imaginative bend. Rough brushstrokes, rich texture, and raw emotive color (*2*) invoked the spiritual underpinnings of cultural traditions rather than the traditions themselves. The paintings contained strong intuitive structure and contemporary elegance.

Day’s “cultural memory” refuted presumptions that the California Indians were vanishing, and he was heralded for his inspiring presence during the revitalization of California Indian arts in the 1960s and 1970s. His artistic contributions were celebrated in “Memory and Imagination,” a major exhibit organized by the Oakland Museum of California in 1997. Day’s works are an authoritative tribute to Native American heritage and its focus on the spiritual connection between humanity and nature (*3*).

Infectious diseases (from smallpox and plague to tuberculosis and influenza) featured in many of the Indian legends whose essence Day sought to preserve (*4*). Blending the dangerous with the supernatural, these legends weaved historical accounts into tales of mystery, medicine, and magic and celebrated the creative spirit with which Native tribes approached disease survival. One painting, The Burning of the Roadhouse, commemorated therapeutic burning of dwellings to rid them of disease; another, Sunflower Remedy, portrayed a dazzling sunflower shielding a child from tuberculosis.

The Water Test, on this cover of Emerging Infectious Diseases, is a culmination of Day’s Native Indian and artistic philosophy: everything is interconnected and imbued with spiritual energy that can be positive or negative. In this symbolic composition, human presence is in center stage. The pastoral scene, afloat in nature, is spare and horizontal but full of vitality. Water, a critical element providing not only physical but also spiritual sustenance, is set off by dramatic earth tones, balanced on the left by a thriving tree and in the diagonal center by a fallen one, which (as if charged by unknown energy) stretches to infinity. A man leans over the water, perhaps to test if it is clear enough to drink or warm enough to get into. Distracted by his reflection, he assumes a narcissistic posture and smiles at his robust image, his own character now being playfully tested by the water. His body is perfectly balanced and in control, but his relationship with the environment seems ambiguous. The water bank is teeming with oversized centipedes, some lurking in bellicose conference under a rock, some venturing out for prey. Their proximity, inflammatory colors, and poised poisoned fangs exude hostility.

The realistic encounter of man and water is embroidered with fantasy. The water contains invisible seeds of harm. The artist, acknowledging that the man’s water test is as vain and elusive as his reflected image, pulls out of the water and into the foreground the centipedes, crude indicators of harm amplified and exposed to the naked eye.

Our water tests are more refined, but they still seek indicators of harm. While we search for better evidence of their presence, harmful critters remain hidden. Standard plate counts or coliform counts are reasonable predictors of microbial presence, but as we peer deeply into our water, other microbes—noroviruses, Giardia, Cryptosporidium—continue to elude us, testing our essential drinking water and our survival.
